# Elastic AlignedSENSE for Dynamic MR Reconstruction: A Proof of Concept in Cardiac Cine

**DOI:** 10.3390/e23050555

**Published:** 2021-04-29

**Authors:** Alejandro Godino-Moya, Rosa-María Menchón-Lara, Marcos Martín-Fernández, Claudia Prieto, Carlos Alberola-López

**Affiliations:** 1 Laboratorio de Procesado de Imagen, E.T.S.I. Telecomunicación, Universidad de Valladolid, Paseo Belén 15, 47011 Valladolid, Spain; rmenchon@lpi.tel.uva.es (R.-M.M.-L.); marcma@tel.uva.es (M.M.-F.); caralb@tel.uva.es (C.A.-L.); 2 School of Biomedical Engineering and Imaging Sciences, King’s College London, London SE1 7EH, UK; claudia.prieto@kcl.ac.uk; 3 School of Engineering, Pontificia Universidad Catolica de Chile, Santiago 4860, Chile

**Keywords:** non-rigid registration, elastic motion

## Abstract

Numerous methods in the extensive literature on magnetic resonance imaging (MRI) reconstruction exploit temporal redundancy to accelerate cardiac cine. Some of them include motion compensation, which involves high computational costs and long runtimes. In this work, we proposed a method—elastic alignedSENSE (EAS)—for the direct reconstruction of a motion-free image plus a set of nonrigid deformations to reconstruct a 2D cardiac sequence. The feasibility of the proposed approach was tested in 2D Cartesian and golden radial multi-coil breath-hold cardiac cine acquisitions. The proposed approach was compared against parallel imaging compressed sense (sPICS) and group-wise motion corrected compressed sense (GWCS) reconstructions. EAS provides better results on objective measures with considerable less runtime when an acceleration factor is higher than 10×. Subjective assessment of an expert, however, invited proposing the combination of EAS and GWCS as a preferable alternative to GWCS or EAS in isolation.

## 1. Introduction

Motion is the main source of artifacts in MRI, especially in modalities with long scan times or when imaging moving organs, as in the case of cardiac cine [1]. In this modality, the cardiac cycle is divided into intervals of short duration, so-called cardiac phases, in which motion can be considered negligible; the k-space is acquired sequentially, and every profile is classified into the corresponding phase. To this end, navigator signals are required, which can be simultaneously recorded (gated acquisitions) [2] or estimated directly from the data (self-gated acquisitions) [3,4,5,6,7].

These approaches are commonly used in clinical practice; however, MRI is slow, limiting the spatial resolution and coverage achieved with these methods. Numerous methods have been proposed to accelerate cardiac cine acquisitions by exploiting temporal redundancy along the different cardiac phases [8,9,10]. A complete review on accelerated cardiac cine reconstruction approaches can be found in [1]. A common approach is to incorporate motion information to regularize the reconstruction problem in order to promote signal sparsity and achieve higher acceleration factors (AFs), also known as reduction factors (Rs) [11,12,13,14,15,16]. A different approach is based on including a motion model not directly in the data consistency term (as opposed to the regularization term) to improve reconstruction [17,18]. In this work, we focused on the latter techniques.

Motion can be incorporated into the forward model of MR acquisition. The method proposed in [17] is able to handle elastic motion models. It is assumed that a corrupted image comes from a general matrix equation, the inversion of which provides the ideal image of the scanned object. This formulation can also be used to estimate motion using only partial spectral information, as shown in the alignedSENSE approach [18], where the authors reconstructed a static, multi-shot, 3D MRI brain volume subject to rigid motion between shots. Estimated motion is incorporated into the reconstruction model in an iterative manner to obtain a motion-free image. Their method does not assume any prior model for the image to be reconstructed, does not make use of external sensors, and does not require modifications in the acquisition sequence [18]. The downside is that only rigid motion is tackled; this type of motion, however, is not suitable for deformable organs, such as the heart. Nevertheless, this idea of creating a single motion-free image (say, a pattern image) that is deformed to match the measured data may be of great interest to build a cardiac cine reconstruction framework, as long as elastic motion is incorporated.

This work focused on the alignedSENSE formulation, which we extended to the elastic case, and we employed the 2D cardiac cine MRI reconstruction problem as a proof of concept.

## 2. Materials and Methods

### 2.1. Materials

We used 2D Cartesian data from fully sampled dynamic cine breath-hold (BH) gated acquisitions performed on 7 healthy subjects in a 1.5 T Philips scanner with a bSSFP sequence. Some relevant parameters of the acquisitions included flip-angle 60°, TR/TE = 3/1.5 ms, spatial resolution 2 × 2 mm^2^, slice thickness 8 mm, 20 cardiac phases, and FOV 320 × 320 mm^2^.

2D whole-heart single-breath-hold acquisitions with a golden radial trajectory and 32-element cardiac coil were performed on the same subjects on a 1.5 Philips scanner with a bSSFP sequence. Some relevant parameters included TR/TE = 2.9 ms/1.44 ms, flip-angle 60°, spatial resolution 2 × 2 mm^2^, slice thickness 8 mm, and FOV 320 × 320 mm^2^. Twelve short-axis slices were acquired in a single 9.23 s breath-hold scan.

### 2.2. Reconstruction Problem: Elastic AlignedSENSE

The alignedSENSE formulation for parallel multi-shot imaging can be written in matrix form as follows [18]:

(1) $$\left\{\tilde{m},\tilde{\Theta }\right\}=\underset{m,\Theta }{\mathrm{arg min}}{∥A𝓕SU_{\Theta }m-y∥}^{2}$$

where y denotes the acquired k-space data, m is the image to be reconstructed (both in vector form), $U_{\Theta }$ the rigid motion transformation matrix, S the coil sensitivity map, *𝓕* the Fourier transform, and A the sampling matrix. The proposed elastic alignedSENSE (EAS) extends the approach in Equation (1) to consider nonrigid deformations, referred to as $T_{\Theta }$. This is achieved by using a 2D free-form deformation (FFD) model based on B-splines [19] to describe the *N* nonrigid deformations, with *N* the number of cardiac phases, i.e., the number of frames. FFDs are based on a parametric model that deforms an object by manipulating a mesh of control points $\left\{u_{k}|1\leq k\leq M\right\}$. Here, we considered the mesh of control points common for all the frames. The transformation parameters are referred to as $\Theta =\left\{\Theta_{n}|1\leq n\leq N\right\}$ with $\Theta_{n}=\left\{\theta_{n,u_{k}}\right\}$, $\theta_{n,u_{k}}\in R^{2}$. Control point positions are denoted by $p_{k}=\left(p_{k,1},p_{k,2}\right)$, 1≤k≤M, and are uniformly spaced in coordinate system $X_{n}\subset R^{2}$; as indicated, the *M* control point positions will coincide for the *N* frames as well as their coordinate spaces, i.e., ${𝓧}_{n}\equiv X,1\leq n\leq N$, although deformations of course differ. Point $x=(x_{1},x_{2})\in X$ is transformed by the *n*-th transformation as:

(2) $$x_{n}=T_{\Theta_{n}}\left(x\right)=x+\sum_{u_{k}\in N\left(x\right)}\left[\prod_{l=1}^{2}B_{E}\left(\frac{x_{l}-p_{k,l}}{\Delta_{l}}\right)\right]\theta_{n,u_{k}}$$

with $\Delta_{l}$ the spacing between two consecutive points along dimension *l* and $B_{E}$ representing the uniform B-spline function of order *E* [20]. We chose E=3, since those B-splines showed a good balance between smoothness and the support region [21,22]. Since B-spline functions have compact support, only the control points $u_{k}$ within the neighborhood N(x) of point x enter the summation. Notice that, in an abuse of notation, $T_{\Theta }$ may have different meanings depending on the context; in $T_{\Theta }m$, $T_{\Theta }$ represents the interpolation coefficient matrix that allows us to obtain a set of *N* transformed images from image m; complementarily, $T_{\Theta }\left(x\right)$ represents a 2N component vector of the transformed positions of point x from each of the *N* images. $T_{\Theta_{n}}$ is used similarly for the *n*-th transformation. Finally, when we need to highlight operations in the transformation temporal dimension, we employ the notation $T_{\Theta }\left(x,t\right)$, where *t* takes one temporal value {1,…,N}, as appropriate.

Thus, the proposed EAS reconstruction problem is formulated as follows:

(3a) $$\left\{\tilde{m},\tilde{\Theta }\right\}=\underset{m,\Theta }{\mathrm{arg min}}{∥A𝓕ST_{\Theta }m-y∥}^{2}+\lambda {∥\nabla_{x}m∥}^{2}+R_{1}\left(\Theta \right)$$

(3b) $$R_{1}\left(\Theta \right)=\sum_{n=1}^{N}\sum_{x\in X}\left(\omega_{1}{\left|\frac{\partial T_{\Theta }\left(x,t\right)}{\partial t}\right|}_{t=n}^{2}+\omega_{2}{\left|\frac{\partial^{2}T_{\Theta }\left(x,t\right)}{\partial t^{2}}\right|}_{t=n}^{2}\right)$$

where $\nabla_{x}$ denotes the spatial total variation (spTV), which promotes the removal of artifacts in the pattern image, whereas the regularization term $R_{1}\left(\Theta \right)$ favors smoothness in the temporal trajectory, since reconstructions may present some tremor. In Equation (3b), $\omega_{1}$ and $\omega_{2}$ are regularization parameters to be set, and derivatives are approximated by temporal finite differences.

The joint problem in Equation (3a) is solved in an alternating fashion by iteratively solving the two following subproblems:

(4a) $$\tilde{m}=\underset{m}{\mathrm{arg min}}{∥A𝓕ST_{\tilde{\Theta }}m-y∥}^{2}+\lambda {∥\nabla_{x}m∥}^{2}$$

(4b) $$\tilde{\Theta }=\underset{\Theta }{\mathrm{arg min}}{∥A𝓕ST_{\Theta }\tilde{m}-y∥}^{2}+R_{1}\left(\Theta \right)$$

**Figure 1:**
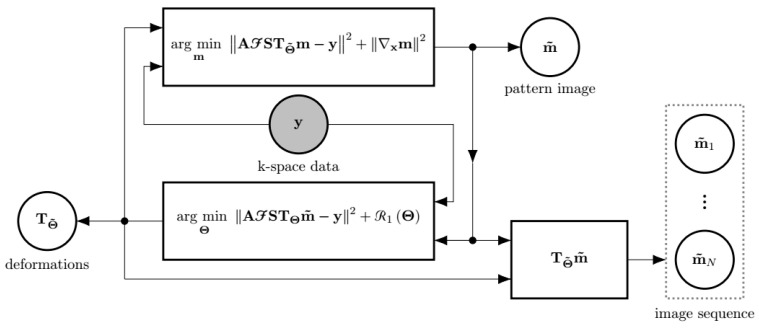
The scheme of the EAS reconstruction as an alternating minimization approach. If the deformations $T_{\Theta }$ are assumed to be known, the best possible m in terms of fidelity to the measured data y can be obtained. Likewise, assuming m to be known, the best possible $T_{\Theta }$ can be obtained. The final image sequence is obtained by applying each of the transformations $T_{\tilde{\Theta }}$ to the pattern image $\tilde{m}$. The input to the reconstruction method is the shaded circle. Outputs are enclosed by a dashed line rectangle.

The first subproblem (Equation (4a)) is referred to as the image subproblem—since its solution is a new image pattern—whereas the second (Equation (4b)) is referred to as the deformation subproblem, since its solution is a new set of deformations. The loop starts by solving the image subproblem (Equation (4a)), considering that there is no transformation, i.e., $T_{\Theta }$ equals the identity, so that an initial pattern image $m_{0}$ can be obtained. After that, the deformation subproblem is fed with $m_{0}$, and the loop can continue as expected (see Figure 1).

The image subproblem (Equation (4a)) was solved by means of a conjugate gradient algorithm [17] and the deformation subproblem (Equation (4b)) by means of a nonlinear conjugate gradient algorithm with backtracking line search [23]. Note that the pattern image arises as a result from the optimization subproblem in Equation (4a) and does not necessarily correspond to any pre-selected cardiac phase.

The extension of EAS to radial trajectories is rather straightforward, since we only needed to substitute the regular FFT for the non-uniform FFT (NUFFT) [24] in Equation (3a):

(5) $$\left\{\tilde{m},\tilde{\Theta }\right\}=\underset{m,\Theta }{\mathrm{arg min}}{∥𝓖T_{\Theta }m-y∥}^{2}+\lambda {∥\nabla_{x}m∥}^{2}+R_{1}\left(\Theta \right)$$

where the operator 𝓖 includes sensitivity coil maps, as well as gridding, NUFFT, and subsampling operations. NUFFT was computed by using the existing implementation for a GPU in [25] (gpuNUFFT).

### 2.3. Methods Used for Performance Comparison

The proposed method was compared with a simple parallel imaging compressed sense formulation (sPICS) [10]:

(6) $$\tilde{m}=\underset{m}{\mathrm{arg min}}{∥A𝓕Sm-y∥}^{2}+\lambda {∥\nabla_{t}m∥}_{\ell 1}$$

where $\nabla_{t}$ represents the temporal total variation (tTV) operator. The proposed EAS approach was also compared with the group-wise (GW) motion-compensated (MC) compressed sense algorithm (GWCS) [16]:

(7) $$\tilde{m}=\underset{m}{\mathrm{arg min}}{∥A𝓕Sm-y∥}^{2}+\lambda {∥m∥}_{J_{T_{\Theta }}}$$

where ${∥m∥}_{J_{T_{\Theta }}}$ is the Jacobian weighted tTV regularization term described in [16]; $T_{\Theta }$ stands for the GW-MC operator, which fosters sparsity in the temporal direction by mapping each frame in m to a common reference. Hence, we first performed a regular reconstruction solving Equation (6) from which the transformation was estimated. The registration metric used was the sum of squared differences (SSD) between the images in the sequence and the common reference; for the SSD, the optimum reference is known to be the average of the registered images [26]. Therefore, transformation parameters are obtained by:

(8) $$\tilde{\Theta }=\underset{\Theta }{\mathrm{arg min}}\sum_{n=1}^{N}\sum_{x\in X_{cr}}{\left(m_{n}\left(T_{\Theta }\left(x,n\right)\right)-\frac{1}{N}\sum_{k=1}^{N}m_{k}\left(T_{\Theta }\left(x,k\right)\right)\right)}^{2}+R_{2}\left(\Theta \right)$$

where *n* and *k* represent frame indices and, as before, *N* is the number of frames; $X_{cr}$ denotes the coordinate space in which the common reference is defined, and $m_{n}\left(x\right)$ denotes the *n*-th frame at point x. $R_{2}\left(\Theta \right)$ is a regularization term that promotes the local invertibility of the deformations, which can be expressed as follows:

(9) $$R_{2}\left(\Theta \right)=\sum_{n=1}^{N}\sum_{x\in X_{cr}}\gamma_{1}{∥\nabla_{x}^{2}T_{\Theta }\left(x,n\right)∥}^{2}+\gamma_{2}{∥\nabla_{t}^{2}T_{\Theta }\left(x,n\right)∥}^{2}$$

where $\gamma_{1}$ and $\gamma_{2}$ are regularization parameters, the values of which were set as in [15]. Both Equations (6) and (7) were solved using NESTA [27].

**Figure 2:**
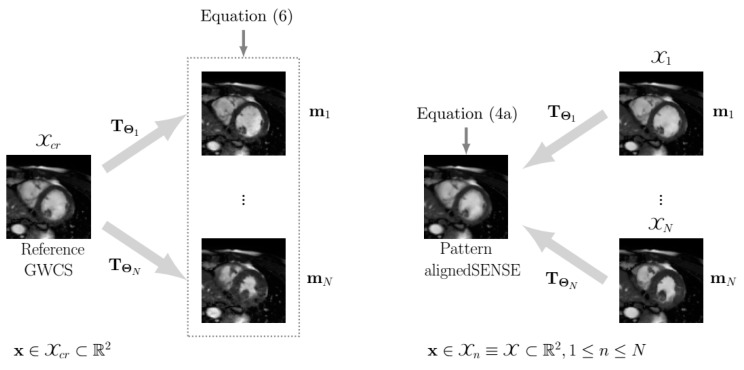
The scheme of spatial transformations in GWCS (**left**) and EAS (**right**) for 2D cardiac cine MRI. **Left**: points to be transformed $x\in X_{cr}\subset R^{2}$ are defined on the common reference coordinate space. **Right**: points to be transformed $x\in X_{n}\equiv X\subset R^{2}$, 1≤n≤N are defined on each image coordinate space, which coincides for all images.

Notice that the registrations in GWCS and EAS, although quite similar in conception and notation, differ in two relevant elements:

1. Transformations are defined in opposite directions, as illustrated in Figure 2. In GWCS, the coordinate space $X_{cr}\subset R^{2}$ is defined in the common reference image, and each frame $m_{n}$ (1≤n≤N, *N* being the number of frames) is transformed so that it fits into such $X_{cr}$, i.e., we calculated $m_{n}\left({𝓣}_{\Theta_{n}}\left(x\right)\right)$ with $x\in X_{cr}$. Thus, in the optimization problem described in Equation (8), we aimed to find that $m_{p}\left({𝓣}_{\Theta_{p}}\left(x\right)\right)≅m_{q}\left({𝓣}_{\Theta_{q}}\left(x\right)\right)$, with p≠q. In the case of EAS, the coordinate space $X_{n}\subset R^{2}$ is defined in each frame $m_{n}$—and coincides for all frames ($X_{n}\equiv X$, 1≤n≤N)—so that each frame $m_{n}$ is a deformed version of the pattern image m, i.e., $m_{n}=m\left(T_{\Theta_{n}}\left(x\right)\right)$. In summary, the transformations have their origin in the space in which the coordinate system is defined, and the direction is the opposite of what “common sense” dictates. The reason for this is because the transformation defined in that way makes the underlying interpolation process more convenient.
2. The common reference image in GWCS is the average of the registered images, following [26], while in EAS, the reference arises as a result of the optimization subproblem in Equation (4a), which is transformed to create the images of the final sequence and does not necessarily correspond to any pre-selected cardiac phase.

### 2.4. Combination of Elastic AlignedSENSE and Group-Wise Motion-Compensated Compressed Sensing

**Figure 3:**
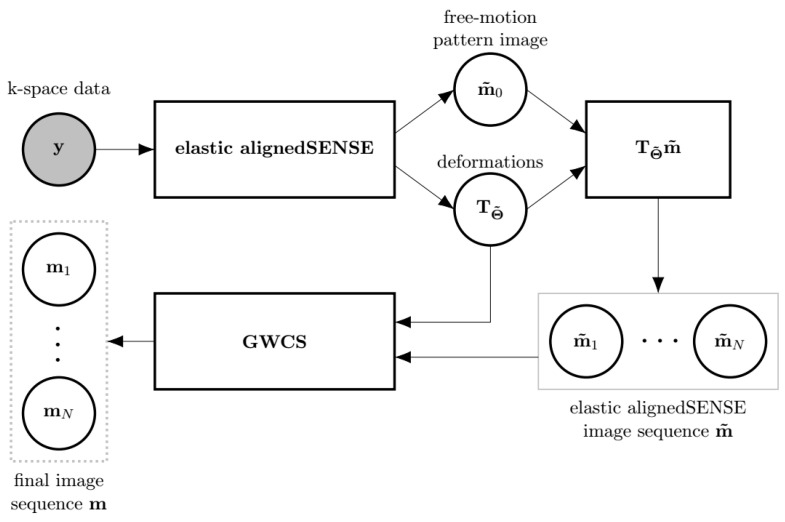
The scheme of the MIX reconstruction method as a combination of, at least, two EAS phases followed by a GWCS phase. The output of EAS, $\tilde{m}$ and $T_{\tilde{\Theta }}$, is fed to GWCS. Since EAS provides directly a set of transformations $T_{\tilde{\Theta }}$ that maps the pattern image $m_{0}$ to each cardiac state, there is no need for the registering stage within GWCS. Thus, only the MC stage within GWCS is applied to obtain the final reconstruction. The input to the whole reconstruction method is the shaded circle. Outputs are enclosed by a dashed line rectangle.

EAS may be a method on its own, but it may also be used as an initializer of methods with ME/MC, such as the GWCS approach (Figure 3). Recall from Equations (3a) and (7) that fewer parameters are estimated in the latter with respect to the former, so it is expected that estimations may be stabler with EAS, as least in the first iterations. Therefore, a combination of EAS with GWCS was proposed (referred as to MIX) in an attempt to benefit from the advantages of each.

### 2.5. Performance Analysis and Hyperparameter Selection

**Figure 4:**
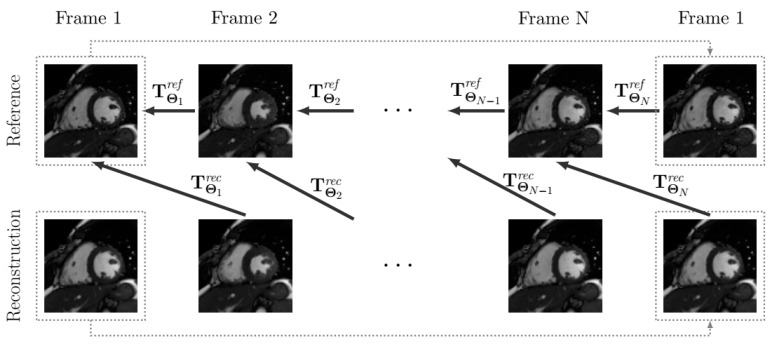
The scheme of the registrations performed for motion quality assessment. Note that a periodic extension is considered (represented with dotted lines), so that the first frame is registered to the last one.

A high-frequency signal-to-error ratio (HFSER) [28] and the structural similarity index (SSIM) [29] have been used for image quality assessment, taking the fully sampled reconstruction as the reference, whenever possible. To measure the quality of motion, we obtained displacement fields by registering the reconstructed sequence and the reference sequence; specifically, the n+1-th frame on the reconstructed sequence was registered to the *n*-th frame on the reference, with *N* the number of frames (we assumed periodicity in the cardiac cycle, so frame N+1 coincides with Frame 1), to obtain the displacement field $D_{\mathrm{rec}}\left(n\right)$, which results from transformation $T_{\Theta_{n}}^{\mathrm{rec}}$, where the latter is calculated by using the motion estimation procedure described above (see Figure 4). Similarly, each frame in the reference sequence was registered to its previous frame in the reference (once again, with periodic extension) to obtain the displacement field $D_{\mathrm{ref}}\left(n\right)$. Finally, the RMSE value between both reference and reconstruction displacement fields is calculated (Equation (10)).

(10) $$\mathrm{RMSE}=\sqrt{\frac{\sum_{n=1}^{N}{∥D_{\mathrm{rec}}\left(n\right)-D_{\mathrm{ref}}\left(n\right)∥}_{F}^{2}}{N}}$$

with ${∥\cdot ∥}_{F}$ the Frobenius norm, considering $D_{\mathrm{ref}}\left(n\right)$ a matrix with dimensions |X|×2, with X the set of reconstructed pixels and |·| the cardinality of a set.

**Figure 5:**
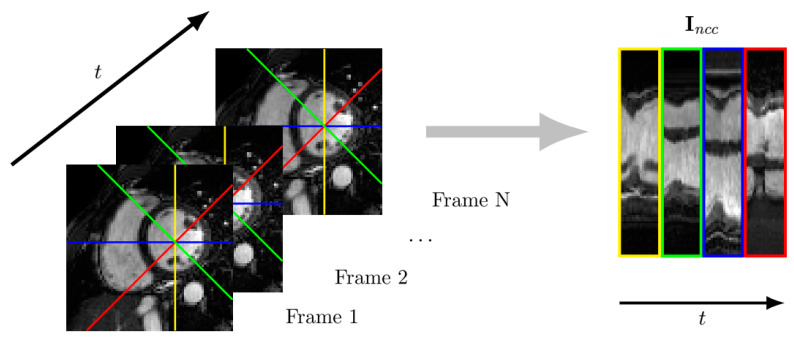
Temporal profiles along radial directions every 45 degrees (the center of which coincides with the center of the left ventricle) are concatenated to form an image. The NCC between such images is used to assess motion quality.

The temporal profiles along radial directions separated 45 degrees were concatenated to form the image $I_{ncc}$ (Figure 5). The normalized crossed correlation (NCC) between such images from both the reconstructed and the reference datasets was also computed as a quality measurement. NCC is defined as:

(11) $$\mathrm{NCC}=\frac{\sum_{x\in X}\left(I_{\mathrm{ncc}}^{\mathrm{ref}}-\bar{I_{\mathrm{ncc}}^{\mathrm{ref}}}\right)\left(I_{\mathrm{ncc}}^{\mathrm{ncrecc}}-\bar{I_{\mathrm{ncc}}^{\mathrm{rec}}}\right)}{\sqrt{\sum_{x\in X}{\left(I_{ncc}^{\mathrm{ref}}-\bar{I_{\mathrm{ncc}}^{\mathrm{ref}}}\right)}^{2}\sum_{x\in X}{\left(I_{\mathrm{ncc}}^{\mathrm{rec}}-\bar{I_{\mathrm{ncc}}^{\mathrm{rec}}}\right)}^{2}}}$$

where $I_{\mathrm{ncc}}^{\mathrm{ref}}$ and $I_{\mathrm{ncc}}^{\mathrm{rec}}$ stand for the $I_{\mathrm{ncc}}$ images obtained from the reference image and the reconstruction, with spatial average values $\bar{I_{\mathrm{ncc}}^{\mathrm{ref}}}$ and $\bar{I_{\mathrm{ncc}}^{\mathrm{rec}}}$, respectively. Since the temporal evolution is accounted for in NCC, this parameter is also useful for motion quality assessment.

Regularization parameters λ from Equation (4a) and $\omega_{1}$ and $\omega_{2}$ from Equation (4b) must be set. To this end, we used the method based on cross-validation described in [30]. This method is grounded on having *K* datasets for training/testing, as well as an IQM to maximize. The procedure to tune the value of a single parameter μ consists of three stages:

1. For each of the *K* datasets, the value of the parameter that maximizes the IQM is determined by sweeping in a range of candidate values; let $\mu_{k}^{ds},1\leq k\leq K$, denote this value for the *k*-th dataset.
2. The K datasets are split into *P* datasets for training and (K−P) for testing. Let $c_{i}$ be the *i*-th training set and $d_{i}$ its corresponding test set, 1≤i≤KP. Let ${\left[c_{i}\right]}_{j}$ denote the index within the set {1,…,K} of the *j*-th element of $c_{i}$, with 1≤j≤P. The purpose of this stage is to determine the optimum parameter for each $c_{i}$. To this end, we accumulated the IQM for all datasets within $c_{i}$, but dataset ${\left[c_{i}\right]}_{j}$, using the parameter $\mu_{{\left[c_{i}\right]}_{j}}^{ds}$ from the previous stage. The optimal value is the one that provides the maximum accumulated IQM out of the *P* accumulated quantities. Let $\mu^{c_{i}}$ denote that value.
3. The final stage pursues finding which of the μci,1≤i≤KP, is the optimum. This is accomplished by calculating the accumulated IQM in the datasets within $d_{i}$, using $\mu^{c_{i}}$; the optimal parameter $\mu_{opt}$ is the value that maximizes this quantity out of the KP accumulated IQM values.

The procedure referred to above can be directly extended to *Q*-component vector parameters by creating a grid of candidate points in the $R^{Q}$ space. For the proposed EAS with variant regularization procedure Q=3. HFSER was used as the IQM to select the regularization parameters.

## 3. Experiments

In this section, we provide an overview of the experiments we conducted. Experimental results themselves are described in Section 4.

### 3.1. Experiment 1: Cartesian Acquisition

The 2D datasets were retrospectively subsampled using the procedure in [12] for different values of the AF and reconstructed by using EAS and MIX. For comparisons, they were also reconstructed using sPICS (Equation (6)) and GWCS (Equation (7)). Using the fully sampled reconstruction as a reference, the HFSER, SSIM, and RMSE between displacement fields, as well as the NCC were computed to measure the performance.

The EAS regularization parameters $\mu =(\lambda ,\omega_{1},\omega_{2})$ were set by using the procedure described in Section 2.5 with K = 7 and P = 4, as in [30]. As for the tentative values for λ, we used six values ranging from 1 to $10^{-3}$ in logarithmic scale, and five values in logarithmic scale from 1 to $10^{-4}$ for $\omega_{i}$ (i=1,2). For sPICS, the regularization parameter was set as in [31] and for GWCS as in [16]. The spacing between control points was set to 3 pixels in both spatial dimensions.

### 3.2. Experiment 2: Radial Acquisition

The 2D golden radial datasets were retrospectively reconstructed by using EAS, with 16 cardiac phases including the maximum of the spokes available per frame, which resulted in an equivalent temporal resolution of 46.4 ms. In this case, there was no availability of a ground truth with which to compare. Thus, the regularization parameters in Equations (5) and (3b) could not be set by applying the method described in Section 2.3. Therefore, some parameter sweeps for λ and $\omega_{i}$ (i=1,2) were performed. Specifically, the parameters varied within the intervals $\lambda \in \left[10^{-7},10^{-4}\right]$, $\omega_{1}\in \left[0,5\times 10^{3}\right]$ and $\omega_{2}\in \left[0,5\times 10^{4}\right]$. The resulting reconstructions were visually inspected, and the parameters were set accordingly. Furthermore, this parameter sweeping revealed that the component of $R_{1}\left(\Theta \right)$ weighted by $\omega_{1}$ (see Equation (3b)) had no perceptible effect in the reconstructions, and therefore, it was discarded.

Finally, the datasets were also reconstructed with iGRASP [32], GWCS, and MIX for comparison purposes. The regularization parameter for iGRASP was set as the authors specified in [33]. For GWCS, the regularization parameter in the MC steps was set to 0.007 after performing some sweeps for λ in the range $\left[10^{-3},10^{-1}\right]$. For the MIX method, the parameters were the same for EAS and GWCS when they acted independently. The spacing between control points was set to 5 pixels in both spatial dimensions.

## 4. Results

### 4.1. Results of Experiment 1

**Figure 6:**
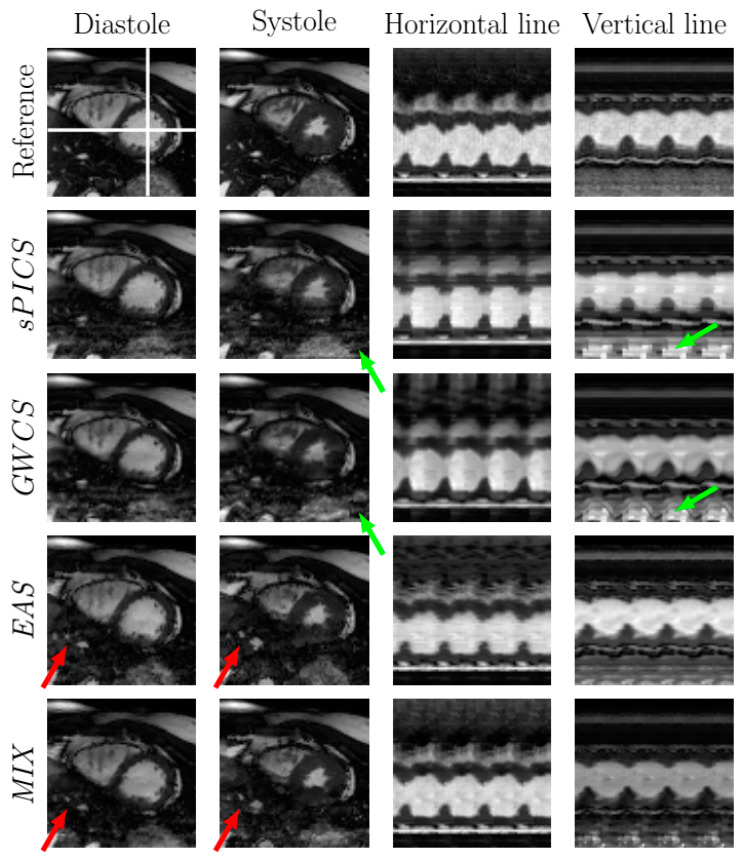
Comparison of EAS and MIX reconstructions with other methods from the literature for a representative case with R=8. The fully sampled reconstruction is included in the top line as a reference. Diastole and systole frames are shown in the two leftmost columns, respectively. Two temporal profiles of the horizontal and vertical lines—marked in the reference image with white lines—are shown in the rightmost columns for all the methods. Arrows point to significant locations.

**Figure 7:**
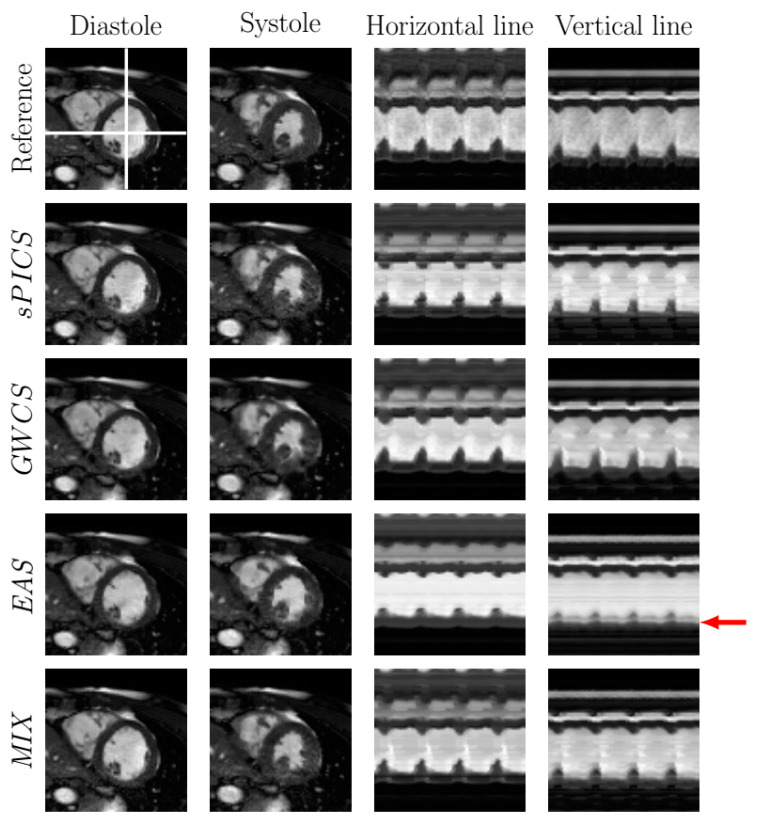
Comparison of EAS and MIX reconstructions with other methods from the literature for a representative case with R=8. The fully sampled reconstruction is included in the top line as a reference. Diastole and systole frames are shown in the two leftmost columns, respectively. Two temporal profiles of the horizontal and vertical lines—marked in the reference image with white lines—are shown in the rightmost columns for all the methods. Arrows point to significant locations.

Figure 6 and Figure 7 display the systole and diastole frames from two representative cases of the 2D dataset reconstructed with EAS and MIX compared with sPICS and GWCS. Two temporal profiles from vertical and horizontal lines are also provided in the two rightmost columns. Both images and temporal profiles from the fully sampled reconstruction are included as a reference in the top line.

**Figure 8:**
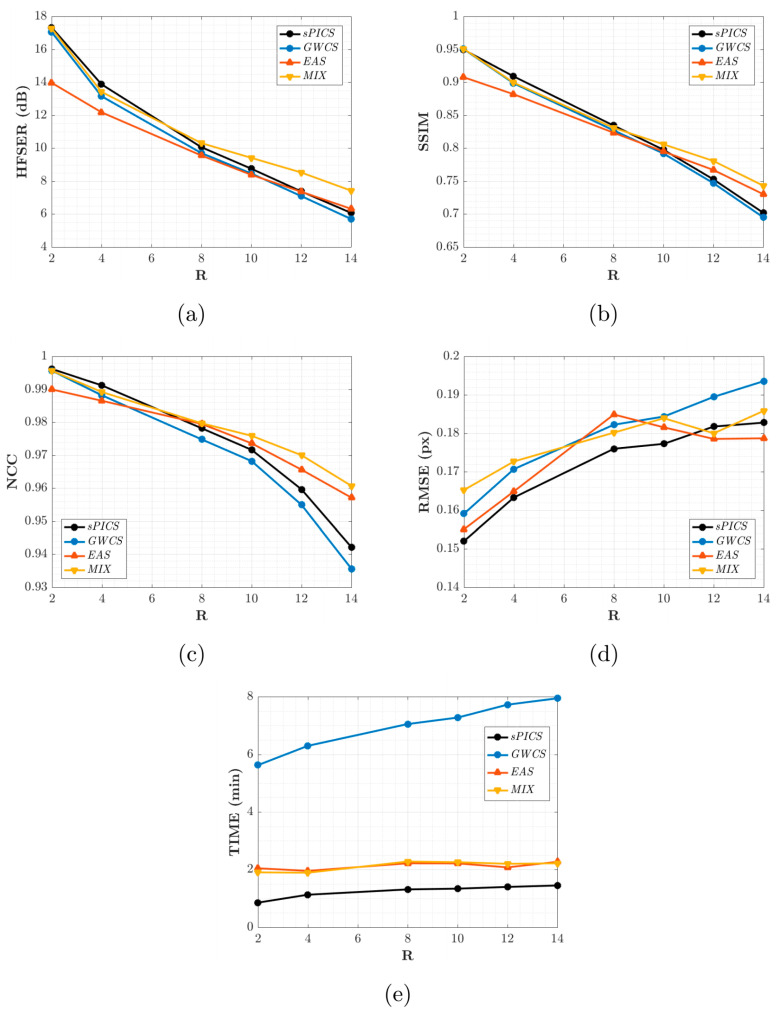
Results for EAS and MIX reconstructions. The average values across slices and volunteers for the HFSER (**a**), SSIM (**b**), NCC (**c**), and RMSE (**d**) and the average time needed to reconstruct one slice (**e**) are provided for different values of *R*.

**Figure 9:**
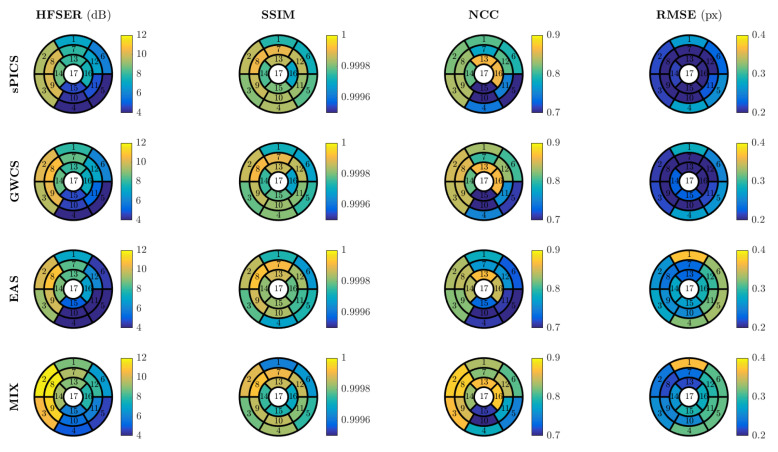
Results for EAS and MIX reconstructions distributed according to the 17-segment AHA model. The average values across volunteers are provided for R=8.

For performance quantification, Figure 8 shows the HFSER (a), SSIM (b), NCC (c), and RMSE (d) averaged across all slices and volunteers, parameterized by *R*, for sPICS, GWCS, EAS, and MIX. The average time needed for reconstructing one slice is also provided in Figure 8e. Figure 9 displays the distribution of these metrics according to the 17-segment AHA model for R=8.

Additionally, a cardiologist—Dr. David Filgueiras-Rama, from the Centro Nacional de Investigaciones Cardiovasculares (CNIC), Spain—was consulted by means of a questionnaire, consisting of 21 videos. Each video displayed a composition of cine reconstructions of the same slice, volunteer, and AF level by applying the different methods to be compared, randomly sorted. The fully sampled image was also included as a reference, and the selected levels of the AF were R=8, R=10, and R=14. The expert was asked to sort the reconstructions in each video according to his perceived quality, giving a score of six to the reconstruction with the best quality and a score of one to the reconstruction with the worst quality. The mean value ± the standard deviation of the scores given by the expert to each reconstruction method are collected in Table 1.

### 4.2. Results of Experiment 2

**Figure 10:**
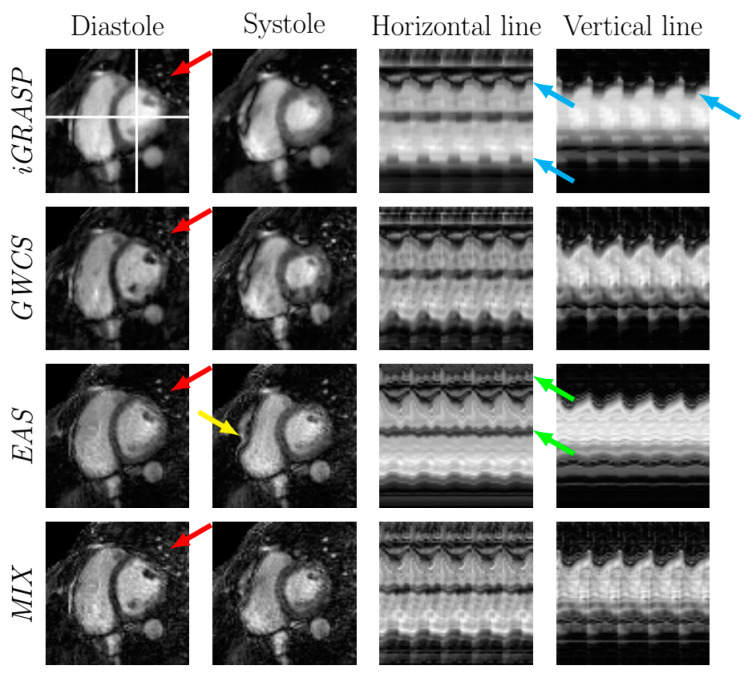
EAS radial reconstructions in comparison with iGRASP and GWCS (R=19.33). Reconstructions from the MIX method are also included. Arrows point to significant locations.

Figure 10 illustrates 2D golden radial reconstructions using EAS in comparison with iGRASP and GWCS for a representative case. The reconstruction using the MIX method is also included. In the two leftmost columns, diastole and systole frames are shown, and in the two rightmost columns, the temporal profiles of both horizontal and vertical lines (marked with white lines in the top left image) are represented. Table 2 shows the average running time needed to reconstruct one slice. The equivalent AF for this example is 19.33.

## 5. Discussion

In terms of image quality, the EAS reconstructions tended to have less subsampling artifacts (Figure 6, green arrows), but they may show some more pronounced blurring, due to the spatial regularization that it is applied in the EAS image subproblem (Figure 7, red arrows). In terms of motion, EAS reconstructions seemed smoother, whereas in the other methods, motion was perceived with sharper transitions, mostly when the AF increased. Nevertheless, in some of the EAS reconstructions, residual fluctuations may also be perceived in the images, as if they were immersed in liquid. This effect probably arises as a consequence of using a B-spline deformation model and sub-optimal regularization parameter tuning.

In addition, EAS tended to show difficulties in homogeneous areas, where registration is known to show worse performance. Since EAS is essentially model based (as opposed to the other methods, which are data driven), the resulting reconstructions had a slight trend of being more static than expected, specifically those in the lateral and basal anterior areas, as revealed by Figure 9, which may lead to a hypokinesia misdiagnosis. An example of this effect is presented in Figure 7. The corresponding EAS reconstruction showed more static inferior/inferior-lateral segments compared to the reference image. This fact manifested itself in the images of the temporal profile of the vertical line (rightmost column of from Figure 7) by a subdued fluctuation in the intensity line due to the contraction of the myocardium (marked with red arrow), which is also supported by Figure 9.

Figure 8 shows that EAS gave rise to values of the metrics more closely related to intensity quality (HFSER, SSIM, and NCC) lower than those provided by the other methods for an AF of less than eight. From this AF value, the metrics began to be approximated, and from R=10, EAS gave rise to slightly higher values than the other ones, GWCS and sPICS.

According to the expert, all the images were useful for diagnosis. He reported the great difficulty of executing the task, since all the images were quite similar and the differences between them were very subtle. He also reported that a homogeneous decision criterion for the entire sample was very difficult to set, since different subtle details had to be accounted for, such us sharpness in trabeculae or papillary muscles, among other structures, depending on the image in every case.

As can be inferred from the results in Table 1, GWCS received, generally speaking, the highest scores. EAS, however, gave rise to motion patterns that the expert was not comfortable with, despite Figure 8 indicating that EAS provided better figures for R≥10. As stated above, the model-based character of EAS seemed to reduce the degrees of freedom in the reconstruction to an extent that other data-driven methods were preferable to the expert eye in terms of motion quality (but not necessarily in terms of image quality). A B-spline parameterization with periodic extension of the FFD, as in [34], would provide an implicit temporal smoothness and enable the use of the B-spline temporal derivative instead of finite differences. However, whether this would be beneficial or would further highlight the model-based character of our solution requires further experimentation.

Hence, due to the fact that the motion provided by GWCS was the preferred expert option, but EAS provided slightly better results with considerably less runtime when R≥10, according to Figure 8, both methods—EAS as an initializer of GWCS—could benefit from their combination, since better motion reconstructions could be achieved with GWCS but at EAS-comparable runtimes. Indeed, this seemed to be supported by Figure 8. MIX values were comparable with those provided by GWCS for lower values of the AF, but they separated when the AF increased, with MIX providing values higher than those from the other methods, including EAS. As far as runtime is concerned, MIX strategies took much less time than GWCS, about two or three times less, reaching the same level as EAS. Therefore, the MIX strategy arose as a competitive reconstruction method to take into account.

Regarding radial trajectories, the iGRASP method provided the most blurred images; the borders of the myocardium were not as defined as in the other methods. In addition, the contraction motion of the heart was reduced, which could be perceived by the smooth intensity waves in the temporal profiles (see the blue arrows in Figure 10). On the contrary, GWCS seemed to reflect heart motion more faithfully, compared to the Cartesian reconstructions from the previous experiments.

EAS also preserved motion, although there was a slight loss of the motion components. This effect could be observed in the intensity fluctuations due to myocardial contraction in the temporal profiles, which were perceived not as prominent as in GWCS. In addition, the torsion motion was not captured by the deformations of EAS, although this torsion was maintained in the Cartesian reconstruction. This was probably due to the fact that the regularization term in the radial EAS deformation subproblem filtered out some motion components, so that a finer tuning of the parameters might be necessary. Despite this, EAS still introduced some residual vibrations in the image sequences, as a consequence of the B-spline model used for deformations. This can be seen in the temporal profiles of Figure 10 as tiny waves in some intensity bands (green arrows). Finally, EAS introduced a non-realistic deformation in the right ventricle (Figure 10, yellow arrows). MIX, on the contrary, managed to capture the torsion motion, and the general motion was perceived as the same quality as GWCS, but with slightly sharper details and less subsampling artifacts.

As far as runtime is concerned, the fastest method was iGRASP, followed by EAS, by about 20 more seconds. The MIX approach reached an intermediate level and took half of the time needed by GWCS, which was the slowest approach, taking almost 6.5 min per slice.

## 6. Conclusions

In this work, we presented the EAS reconstruction method for cardiac cine MRI reconstruction for both Cartesian and radial sampling. This reconstruction method provides a motion-free pattern image together with a set of nonrigid deformations, in which the pattern image is deformed to generate the cardiac cine series. EAS provided slightly better results with considerable less runtime when the AF was higher then 10x. However, the model-based character of the proposal introduced a kind of motion with which experts did not feel comfortable.

The combination of EAS and GWCS as a complete reconstruction method provided images with a better quality in both intensity and motion, or with comparable quality and less computational load, compared to other methods from the literature.

## Figures and Tables

**Table 1:** The mean value ± the standard deviation of the scores given by the expert to each reconstruction method. The scores vary in the range $\left[1,6\right]$, 6 being the method that provides reconstructions with the highest image quality.

	R = 8	R = 10	R = 14
sPICS	5.14±0.69	4.86±0.69	3.43±1.72
GWCS	5.00±1.83	5.71±0.49	4.86±1.68
EAS	2.57±1.13	2.57±0.79	3.29±1.38

**Table 2:** Mean values of the execution times for reconstructing one slice using the EAS and MIX radial approaches in comparison with iGRASP and GWCS.

	Mean Running Time (min)
iGRASP	1.9513
GWCS	6.4263
EAS	2.2940
MIX	3.7792
